# Tissue-specific NETs alter genome organization and regulation even in a heterologous system

**DOI:** 10.1080/19491034.2016.1261230

**Published:** 2017-01-03

**Authors:** Jose I. de las Heras, Nikolaj Zuleger, Dzmitry G. Batrakou, Rafal Czapiewski, Alastair R. W. Kerr, Eric C. Schirmer

**Affiliations:** The Wellcome Trust Centre for Cell Biology, University of Edinburgh, Edinburgh, UK

**Keywords:** DamID, gene regulation, gene position, nuclear envelope, NET, spatial genome organization, tissue specificity

## Abstract

Different cell types exhibit distinct patterns of 3D genome organization that correlate with changes in gene expression in tissue and differentiation systems. Several tissue-specific nuclear envelope transmembrane proteins (NETs) have been found to influence the spatial positioning of genes and chromosomes that normally occurs during tissue differentiation. Here we study 3 such NETs: NET29, NET39, and NET47, which are expressed preferentially in fat, muscle and liver, respectively. We found that even when exogenously expressed in a heterologous system they can specify particular genome organization patterns and alter gene expression. Each NET affected largely different subsets of genes. Notably, the liver-specific NET47 upregulated many genes in HT1080 fibroblast cells that are normally upregulated in hepatogenesis, showing that tissue-specific NETs can favor expression patterns associated with the tissue where the NET is normally expressed. Similarly, global profiling of peripheral chromatin after exogenous expression of these NETs using lamin B1 DamID revealed that each NET affected the nuclear positioning of distinct sets of genomic regions with a significant tissue-specific component. Thus NET influences on genome organization can contribute to gene expression changes associated with differentiation even in the absence of other factors and overt cellular differentiation changes.

## Introduction

The nuclear envelope (NE) contributes significantly to the 3D spatial organization of the genome.^1,2^ There are 2 kinds of NE interactions with the genome: general affinity interactions of NE proteins with heterochromatin^3-5^ and tissue-specific NE transmembrane proteins (NETs) that alter the 3D positioning of specific genes and chromosomes during differentiation.^6,7^ While much work has been done on the former, the latter is relatively newly identified and so there is little understanding of the molecular mechanism for this repositioning and its consequences for gene regulation.

There is no clear consensus on the general question of the relationship between gene position and expression. Some data argues that repositioning drives changes in expression^6,8-10^ while other data argues that the activation state of the gene drives its repositioning.^11^ As the general tendency is for the periphery to be a silencing environment^3,12,13^ and some NETs further recruit silencing enzymes,^14,15^ repositioning a gene to the periphery through a directed mechanism would in theory be followed by the acquisition of silencing marks. At the same time activation/unfolding of a gene removes epigenetically silencing marks and might lower the affinity of a locus for the periphery as some NETs exhibit affinity for silenced chromatin.^5,16^ Similarly designed studies using artificial tethers yielded inconsistent results^8,10,17^ while studies using tissue differentiation systems generally could not distinguish whether observed changes in expression of important repositioning genes were due to the gene repositioning or the many other changing facets of differentiation.^18-21^

The recent identification of several tissue-specific NETs that direct chromosome and gene positioning^6,7,22^ has now enabled the ability to specifically target the repositioning separate from other facets of differentiation. The tissue-specific NETs NET29/Tmem120A (fat), NET39/PPAPDC3 (muscle), and NET47/TM7SF2 (liver) promote peripheral positioning of chromosome 5 in human HT1080 fibroblast cells.^7^ As expected by their tissue-specific expression patterns, these have also been shown to specifically direct chromosome and gene positioning in liver and muscle cells.^6,7^ Moreover, loss of muscle NETs perturbed gene expression during mouse C2C12 myogenesis.^6^ Blocking repositioning by knockdown of these NETs in myotubes revealed that the peripheral association contributes about half of the repression. In contrast, inappropriately contributing the NET function to myoblasts that don't normally express it repositioned the locus to the periphery but did not affect expression, arguing that a combination of position and transcriptional repressors associated with myogenesis is required to achieve the peripheral repression effects.^6^

To determine if the observed changes in gene positioning or expression depended on differentiation, we tested whether the tissue-specific NETs could direct these changes in a heterologous system. Previously we demonstrated that expression of several tissue-specific NETs could promote chromosome repositioning to the NE in heterologous HT1080 fibroblast cancer cells.^7^ Here we have analyzed these cells for corresponding effects of these NETs on gene expression. We find that heterologous expression of liver-, fat- and muscle-specific NETs each yields changes in the expression of different gene subsets. Analysis of global changes in specific gene repositioning yielded a weaker correlation between repositioning and expression than observed in the myogenesis system,^6^ but the same tendencies were observed. These results indicate that while the transcriptional and chromatin-repositioning changes induced by NETs require other factors for successful differentiation, some of the differentiation-associated changes are directed by NETs in the absence of differentiation and even in completely unrelated cell types.

## Results

### Tissue-specific chromosome-repositioning NETs alter gene expression when exogenously expressed in HT1080 fibroblasts

**Figure 1.**
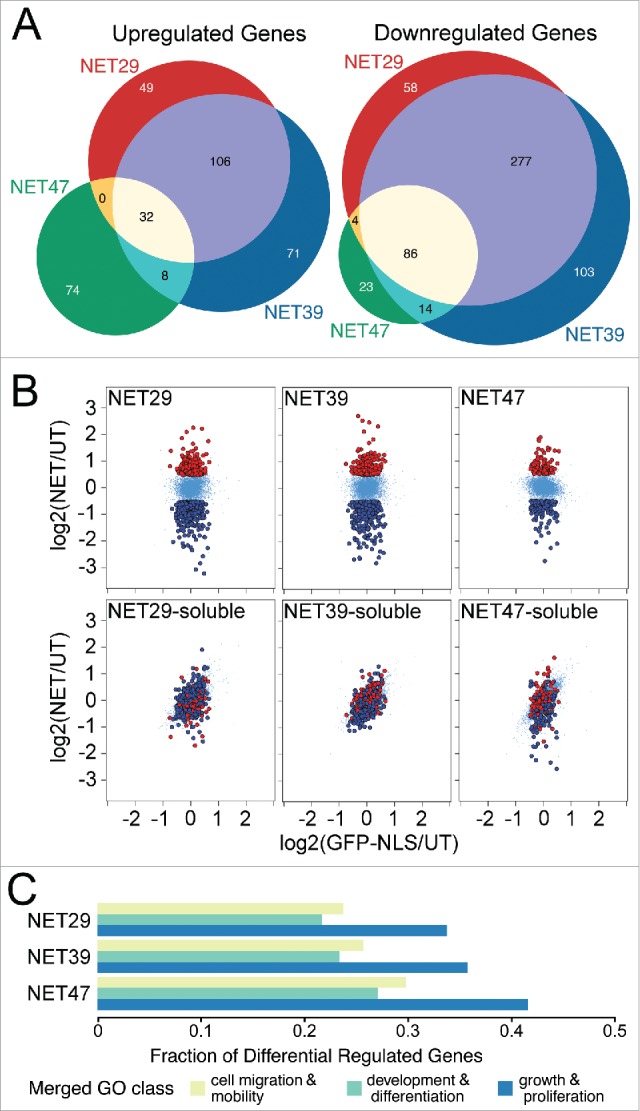
Different chromosome-positioning NETs affect distinct sets of genes when overexpressed in HT1080 cells. (A) Three-way Venn diagram comparing genes that were significantly up- or downregulated in cells that stably expressed each NET. The profiles of the 3 NETs that altered chromosome positioning largely overlapped, but also exhibited a large number of specific differences between them. (B) Anchorage at the NE is required for NET-induced changes in gene expression. HT1080 cells stably expressing soluble NET fragments failed to produce similar gene expression changes as the full-length NETs. These scatterplots compare expression changes for HT1080 cells transfected with GFP fused to each full-length NET (top row) or the corresponding soluble nucleoplasmic fragment (bottom row) on the y-axis, against a GFP-NLS control on the x-axis. Genes highlighted in red or dark blue correspond to genes upregulated or downregulated, respectively, for each full length NET. While the soluble fragments did affect some genes, the differences were smaller in magnitude and the genes were not the same as those affected by the full-length proteins. (C) NETs preferentially affect genes involved in growth, development and differentiation (Table S1B). Gene Ontology (GO) terms associated with those processes were extracted and the fraction of differentially regulated genes shown. Note that due to redundancies in GO terms the total fraction will add up to greater than 1.

We tested whether the NET-induced chromosome repositioning was accompanied by gene regulation changes in the heterologous HT1080 fibroblasts where these NETs are not normally expressed. RNA was prepared from 3 separate passages of HT1080 lines stably expressing GFP-NETs and analyzed on Illumina bead microarrays. NETs 29, 39, and 47 were respectively expressed 2.5, 4.3, and 3.6-fold over endogenous levels by semi-quantitative RT-PCR. Compared to the NLS-GFP control each NET yielded a distinct pattern of overlapping and unique changes in gene expression (Fig. 1A, Supplementary Table S1A).

The proportion of altered genes downregulated by individual NETs ranged from 52% to 70% suggesting an overall tendency toward silencing, possibly as a consequence of peripheral localization. However, comparing the set of genes uniquely regulated by each NET to those overlapping between multiple NETs revealed a slightly more complex situation. Among the upregulated genes, most were uniquely attributed to a single NET: there were 194 genes upregulated by only one of the NETs, and 146 genes shared between at least 2 NETs. In contrast, only 184 of the downregulated genes —roughly a third of the total- were unique for a single NET, and 381 were shared (Fig. 1A).

Some NETs bind transcriptional regulators,^15,25,26^ raising the possibility that a transcriptional regulator rather than the repositioning might be responsible for observed gene expression changes. Transcription regulators would necessarily bind the nucleoplasmic region of NETs. Previous work has demonstrated that when expressed as soluble fragments these nucleoplasmic regions could not reposition chromosomes while the full-length proteins could.^7^ We therefore assayed gene expression changes in HT1080 cells that expressed full-length NET29, NET39 or NET47 (Fig. 1B, top row) or each corresponding soluble nucleoplasmic fragment (Fig. 1B, bottom row). The soluble fragments had some minor effects on gene expression, however they were far smaller in magnitude and only partially overlapping. Together these findings show that both the NET-dependent changes in gene expression and in chromosome/gene repositioning require the NET to be anchored at the NE.

### NET47-directed transcriptional changes reflect Hepatic differentiation changes

Gene ontology (GO) analysis revealed a striking enrichment of genes involved in tissue remodeling, differentiation and proliferation compared with the genome as a whole (Fig. 1C; Supplementary Table S1B). Though these numbers will be to some degree inflated due to redundancy of GO categories, it is clear that NETs affected many such genes. The GO-categories merged for Fig. 1C are listed in Supplementary Table S1C. Enrichment in these GO-categories involved both up- and downregulated genes, but most were downregulated (Supplementary Table S1B). This suggests that a major role of chromosome/gene repositioning is to tightly shut down proliferation in tissues and to further repress alternative differentiation pathways.

**Figure 2.**
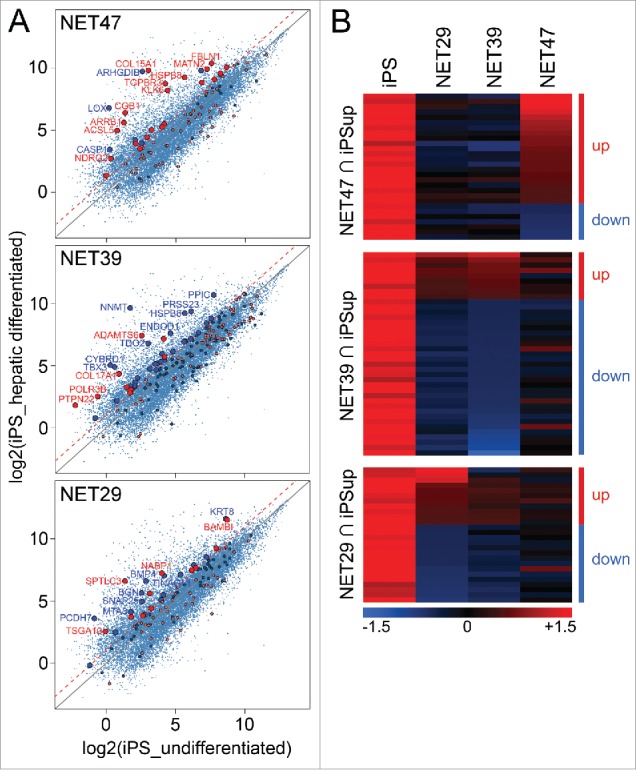
Heterologous expression of NET47 in HT1080 cells yields many gene expression changes characteristic of hepatocyte differentiation. (A) Scatterplot comparison with transcriptional changes seen during iPS hepatic differentiation. A red dotted line indicates the 2-fold upregulation threshold. Genes uniquely regulated by each NET were highlighted in the scatterplots, red if upregulated and blue if downregulated by the NET with respect to the NLS-GFP control. Thus genes above the diagonal were upregulated and below were downregulated in the hepatic system while the color of the spot indicates the direction of its changed regulation by the NET. Though many genes affected by NETs in the HT1080 cells were unchanged in the hepatic differentiation system, the more liver-specific NET47 has a larger proportion of genes that were upregulated in both systems than the other 2 NETs. Those with >2-fold upregulation are enlarged. (B) Heatmap of intersects between genes upregulated in the iPS hepatogenesis system and the genes altered in expression by NETs in HT1080 fibroblasts. For each NET a separate heatmap is given for the genes changing in both the iPS system and that NET in the HT1080 fibroblasts and then the genes affected by the other NETs for that subset are also compared in the adjacent columns. The up and down designations apply to the NET defining each set/ heatmap. The more liver-specific NET47 contains a larger proportion of genes changing in the same direction as the iPS hepatic differentiation system while NET39 has few.

As NET47 knockout mice exhibited loss of expression of several liver genes,^27^ the microarray data for NET expression in HT1080 cells was analyzed for positive changes reflecting liver differentiation. To do so, microarray data from a study differentiating iPS cells into hepatic cells (GEO accession GSE14897) were extracted and the gene changes plotted. Genes uniquely changed in expression by NETs that were in the hepatic differentiation data sets were highlighted on the hepatic plots and colored red if the NET upregulated the gene and blue if the NET downregulated the gene (Fig. 2A). NET47, which was preferentially expressed in liver, had several upregulated genes in the HT1080 data sets that overlapped with upregulated genes in hepatic differentiation *i.e.* red spots appearing above the dotted 2-fold upregulation line. The visible trend for more upregulated genes in differentiation matching with genes upregulated in the NET47 HT1080 data sets was further supported by the probability of achieving this distribution randomly being only 8% (Kolmogorov-Smirnov (KS) test), which is remarkable considering that the HT1080 fibrosarcoma cells likely express few, if any, liver-specific accessory factors. Notably the genes involved in hepatic differentiation had virtually no overlap with those affected by the soluble fragments (Supplementary Table S1A). The genes affected tended to be important for hepatic differentiation and liver function. The importance of collagen (*COL15A1*) and acyl-coenzyme A synthetase (*ACSL5*) are obvious for liver function. TGF-β signaling (*TGFBR3*), arrestin β (*ARRB1*), kallikrein peptidase (*KLK6*), procollagen C-proteinase enhancer protein (*PCOLCE*), and matrilin (*MATN2*) are all important for liver differentiation and/or regeneration.^28-32^

The overlap between genes upregulated during hepatic differentiation and genes upregulated due to NET overexpression was only observed for the liver-specific NET47, and not for NET39 (muscle) or NET29 (fat). Interestingly, for NET39 the overlap between genes differentially expressed and those upregulated in hepatic differentiation strongly highlighted genes that were downregulated by the overexpression of the NET (blue spots, KS p < 0.01). Again many of the most affected genes are notably important for liver function such as cytochrome B (*CYBRD1*), peptidyl-prolyl isomerase C (*PPIC*) and tryptophan 2,3-dioxygenase (*TDO2*) or liver differentiation such as the T-box transcription factor TBX3.^33^ The effects of NET29 were milder and no significant association was observed in either direction (Fig. 2A). This is more striking when the data are plotted as a heatmap to show both the relative intensities of the changes as well as their relative numbers (Fig. 2B). The liver-specific NET47 upregulated 21 genes and downregulated only 7 genes. In contrast, the muscle-specific NET39 favored downregulation (30 genes) over upregulation (9 genes). The fat-specific NET29 showed an intermediate effect (11 and 15 genes being up- or downregulated respectively). The more strongly upregulated genes were present in the NET47 set while the more strongly downregulated genes were found in the NET39 set, as evident by the color intensity on the heatmaps.

### Global determination of genes changing at the periphery with exogenous NET expression

Previous work demonstrated that, for the 2 chromosomes tested, all 3 NETs can reposition chromosome 5 and NET29 and NET39 could also reposition chromosome 13 in the HT1080 cells.^7^ Yet gene expression changes were distributed throughout most chromosomes (Supplementary Table S1). We therefore used DamID to assay global changes in gene positioning caused by these NETs. DamID, in which a bacterial dam methylase is fused to lamin B1 to uniquely methylate all peripheral DNA,^34^ was employed in control HT1080 fibroblasts and the stable lines overexpressing each tissue-specific NET fused to GFP. As an internal control for chromatin accessibility in each condition, soluble Dam methylase was expressed in parallel cultures. The uniquely methylated DNA was enriched for and sequenced yielding 3.7-fold genome coverage from the control HT1080 cells, 3.4-fold coverage from cells expressing NET29 or NET39, and 7.1-fold coverage from cells expressing NET47 (sequence reads deposited at GEO repository, accession number GSE87228).

**Figure 3.**
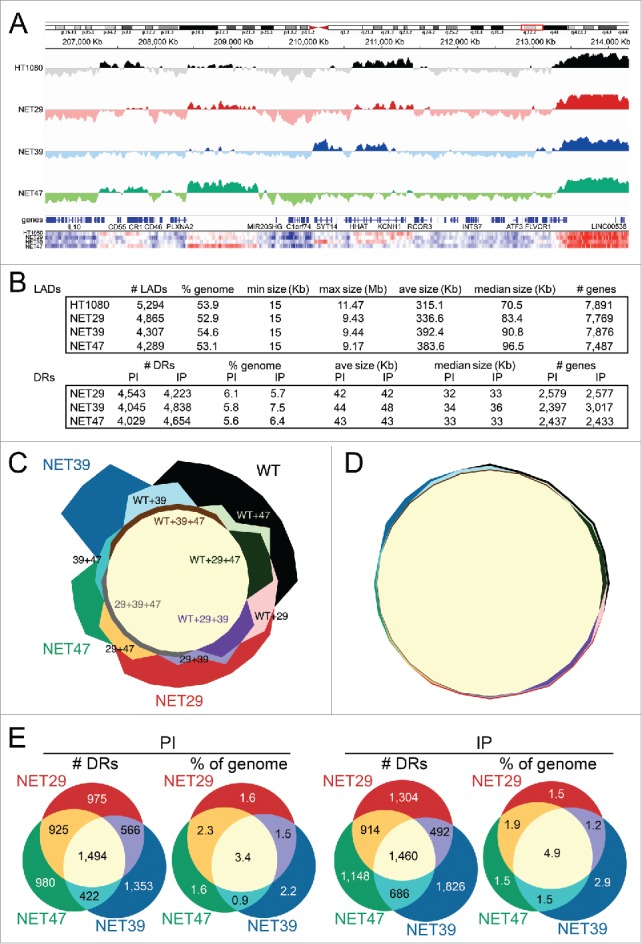
DamID maps of genes repositioned by NET expression in HT1080 fibroblasts. (A) The Log2(Lamin B1-Dam/ soluble Dam) values for the control untransfected and the stable lines expressing each of the 3 NETs are plotted against an 8 Mb region of Chromosome 1. Traces above the line indicate LADs. Bottom heatmaps plot the intensity of signal changes over the same region, using CBS-generated subregion sets (see materials and methods), revealing many additional subtle changes not easily visualized in the standard DamID traces. (B) Summary of LADs (top) and differential regions (DRs, bottom) between control HT1080 and HT1080 overexpressing one of 3 NETs indicating number of LADs/DRs, genome coverage, size range, average and median size, as well as number of genes overlapping those regions. DRs are classified as “IP” or “PI” which denote regions that become peripheral or lose peripheral association, respectively, upon overexpression of each NET. (C) Four-way proportional Chow Ruskey diagram comparing the number of LAD clusters and their intersects among HT1080 control (WT) and HT1080 cells overexpressing NET29, NET39 or NET47. The central pale yellow area corresponds to LAD clusters shared by all 4 conditions. A significant number of LAD clusters are specific for a single condition. (D) Four-way proportional Chow-Ruskey diagram comparing the genomic coverage of LAD clusters and their intersects among HT1080 control (WT) and HT1080 cells overexpressing NET29, NET39 or NET47. This panel is matched to and color-coded identically to panel C so that the small intersecting regions can be identified without labels. Although there are many LAD clusters unique to each condition, they represent a small proportion of the genome. (E) Three-way proportional Venn diagram comparing the number of differential regions (DRs) and genomic coverage (% of genome) changing with overexpression of each NET. PI (DRs moving away from the periphery) diagrams are shown on the left and IP (DRs moving toward the periphery) diagrams on the right.

Log2(Lamin B1-Dam/ soluble Dam) ratios were generated for the numbers of sequencing reads across the genome and plotted to identify lamina-associated domains (LADs) at the nuclear periphery. This ratio represents, in essence, the likelihood that a particular region is associated with the NE averaged across the whole sample cell population over 2–3 cell divisions. An example DamID trace is shown in Fig. 3A. Regions above the midline represent LADs. In the roughly 8 Mb region shown the patterns were shared in some areas and differed in others with some unique changes driven by individual NETs. This clearly shows that each NET has distinct effects. For example, the LAD encompassing the *CR2-CR1-CR1L* gene cluster is observed in the control cells and the NET47 overexpressing cells, but is diminished in the NET29 overexpressing cells and disappears entirely in the NET39 overexpressing cells (Fig. 3A). These results suggest 2 kinds of LADs. The strongest intensity LADs barely change between conditions and tend to be long, often several Mbp long. The rest of the LADs exhibit a range of intensities and tend to be shorter, often encompassing a single gene or gene cluster as shown in Fig. 3A. Most of the LADs that change between conditions are from this latter group and have often been referred to as ‘facultative’ LADs in the literature.^35^

Plotting the relative intensities along the trace as a heatmap revealed additional subtle differences between the NETs in their effects on peripherally associated genome regions (Fig. 3A, bottom). Within the defined LADs there were often smaller regions exhibiting signal intensity changes for Log2(lamin B1-Dam/ soluble Dam) ratios, consistent with previous reports that a binary LAD/ non-LAD definition is insufficient to identify all changing regions due to NET function.^6,35^ Thus, with the view that the Log2(lamin B1-Dam/ soluble Dam) signal represents the likelihood of nuclear peripheral localization in the cell population over the duration of the DamID experiment rather than an absolute determination of its LAD/non-LAD nature,^35^ we identified differential regions (DRs) exhibiting significant changes in peripheral association based on LaminB1-DamID signal differences.^6^ A DR that shows a tendency to reposition away from the Periphery toward the Interior is designated PI and, conversely, relocation from the Interior to the Periphery is designated IP. Each NET promoted the PI relocalization for 8–9% of all genes in the genome and promoted the IP relocalization for 8–10% of genes (Fig. 3B).

To facilitate comparison of LADs between conditions, we classified the LADs into clusters of overlapping LADs across conditions, as their boundaries rarely exactly coincide between conditions. Just as gene expression effects exhibited considerable specificity for each NET (Fig. 1A), a similar result was observed regarding LADs. Of 4,624 LAD clusters 1,878 were shared among all 4 conditions and 1,674 were unique for each: 619 for control HT1080 cells, 394 for NET29, 464 for NET39 and 197 for NET47 (Fig. 3C). However, while the number of NET-associated LADs was notable, their genomic coverage was small: only 1.8% of the genome was covered by LADs unique for any single condition (Fig. 3D), indicating that these unique LADs are generally very small. In fact, they ranged between 15 and 352 Kb, with a median size of 28 Kb.

When the specificity of these PI and IP regions was graphed, again the number of changing regions was greater for those specific to individual NETs than changing regions shared by all NETs (Fig. 3E). Unlike gene expression changes that strongly favored downregulation, there was no notable distinction between PI and IP regions with respect to NET specificity. Notably, in contrast to the LADs, when plotted as percentage of the genome the area of the NET-induced DRs overlapping between the 3 NETs did not appreciably change (Fig. 3E).

### The relationship between gene expression changes and repositioning

**Figure 4.**
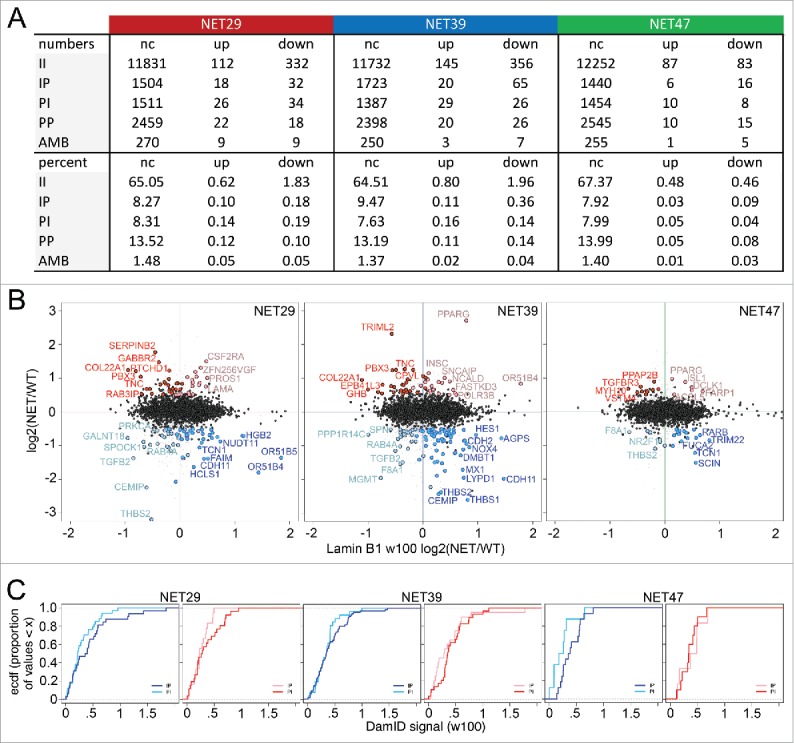
The relationship between NET-induced gene repositioning and expression. (A) Summary of intersect changes between DamID and microarray data. The DamID classes are described as II = stays internal, PP = stays peripheral, PI = shifts away from the periphery, IP = shifts toward the periphery, and AMB = ambiguous *i.e.*, could not be determined. For gene expression data up and down designate upregulated and downregulated after overexpression of each NET, respectively, and ‘nc’ means ‘no change’. (B) Log2(Expression NET-HT1080/ expression control HT1080) gene expression changes are plotted against Log2(DamID NET-HT1080/ DamID control HT1080) of a 100 Kb window centered in each gene for the stable lines expressing each of the 3 NETs. For the colors, red is upregulation and blue is downregulation. The dark red spots correspond to upregulated PI genes and the pale red spots correspond to upregulated IP genes. Dark blue spots indicate downregulated IP genes while pale blue spots indicate downregulated PI genes. All gene expression values are mean average changes of triplicate microarray samples. Data available in Supplementary Table S2. (C) Empirical cumulative distribution frequency (ecdf) plots comparing IP and PI populations within the downregulated (left panel) and upregulated (right panel) genes for each NET. The colors are as in the previous panel: dark/pale blue lines represent downregulated IP/PI genes respectively, while dark/pale red lines represent upregulated PI/IP genes respectively. When a line rises earlier toward the plateau and stays to its left, it means that class of genes have overall a smaller laminB1-DamID signal change (in absolute value) between the control HT1080 cells and cells transfected with a particular NET. For all 3 NETs, the largest changes involved genes that were both downregulated and moved toward the periphery.

In our previous myogenesis study 70% of the genes that were repositioned during myogenesis (both IP and PI) and had altered expression did so in the expected direction *i.e.*, IP genes were downregulated when associated with the periphery while PI genes were upregulated upon release (Fisher's exact test, p < 2.2 × 10^−16^).^6^ In the heterologous system used here, the same tendencies were observed for NET39 and NET47 (67% and 65%, with Fisher's test p < 4.1 × 10^−04^ and 0.06 respectively). For NET29 there were only 50% genes changing position and expression in the expected direction (Fisher's test p < 0.28). The scatterplots show the laminB1-DamID signal plotted against the expression changes (Fig. 4B). Upregulated genes are shown on the top half (strong red for the PI, pale red for IP) and downregulated in the bottom half (strong blue for the IP, pale blue for PI). The stronger colors denote the repositioning and expression changes occurred in the expected direction, while the pale colors indicate the changes occurred in the opposite direction. Interestingly, there were several genes affected by multiple NETs that moved in the opposite direction, *e.g.*, downregulated but moved away from the NE. For example *RAB4A* and *TGFB2* were among the strongest in this category in HT1080 cells overexpressing NET39 or NET29 while *F8A1* was in this category for cells that expressed NET39 or NET47 and *THBS2* in cells expressing NET29 and NET47. For genes that were both upregulated and moved to the periphery there was very little overlap, but interestingly *PPARG* was in this category for both NET39 and NET47, though to different degrees. The list of all genes changing in both directions is given in Supplemental Table S2.

There was a much higher proportion of genes exhibiting changes in the opposite direction than observed previously in the muscle differentiation system.^6^ However, all 3 NETs preferentially downregulated genes that moved to the periphery (IP genes), with NET39 exhibiting both the strongest repositioning and strongest repression (Fig. 4B). To better gauge the difference between these populations, we replotted the data as cumulative distribution plots (Fig. 4C). In all cases the dark blue lines (downregulated IP genes) reach the plateau to the right of (higher DamID signal) the pale blue lines (downregulated PI genes), demonstrating that the expected direction was more prevalent, *i.e.*, downregulated genes that move to the periphery. A similar result was observed for upregulated genes, being more likely to be released from the periphery, except for NET47 possibly due to the low number of changing genes observed. It is interesting that in each case, it is the downregulation/IP association that seems strongest. It is possible that this is a reflection of the nature of the heterologous system in that it probably lacks the relevant tissue-specific transcription factors to activate genes once they move away from the repressive environment of the NE, while as a general property of the nuclear periphery repression may require fewer or no tissue-specific factors.

### Direct comparison of heterologous and differentiation systems

**Figure 5.**
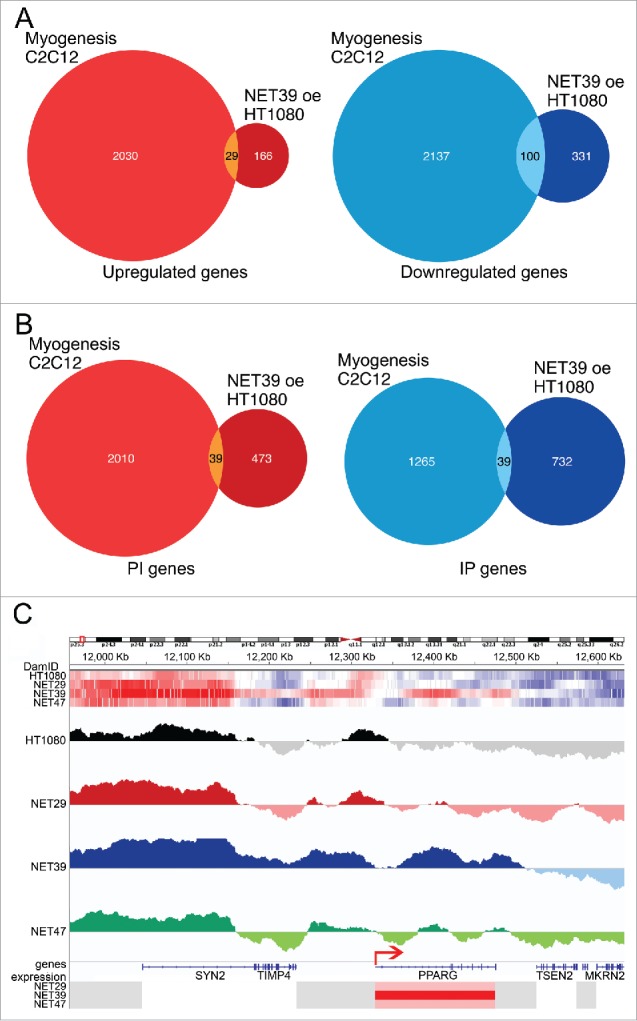
Comparison of NET39-effected changes in the heterologous HT1080 fibroblasts and in myogenesis. (A) Overlap between genes altered in expression (microarray results) by the function of exogenously contributed NET39 in HT1080 fibroblasts and all genes changing during C2C12 *in vitro* myogenesis taken from Robson *et al*.^6^ The Venn diagram on the left shows the genes upregulated by NET39 in HT1080 fibroblasts and upregulated during myogenesis while that on the right shows those downregulated in both cases. Data available in Supplementary Table S3. (B) Overlap between genes altered in position (laminB1-DamID results) by NET39 in HT1080 fibroblasts and all genes changing position during C2C12 *in vitro* myogenesis taken from Robson *et al.*, 2016. The Venn diagram on the left shows the genes moving to the interior (PI) due to NET39 in HT1080 fibroblasts and moving to the interior during myogenesis while that on the right shows those moving to the periphery (IP) in both cases. Data available in Supplementary Table S3. (C) DamID traces displaying the log2(lamin B1-Dam/ soluble Dam) values for the control untransfected and the stable lines expressing each of the 3 NETs are plotted for the neighborhood of *PPARG*. Top heatmaps illustrate the DamID signal over the same region for each NET and control HT1080 cells (blue = low, red = high). Bottom heatmaps indicate the expression changes for the genes in the area (blue = downregulated, red = upregulated). The DamID signal is significantly increased over *PPARG* on the NET39 overexpression cell line, while its expression increases. A small LAD covers the *PPARG* promoter/transcriptional start site (indicated by the red arrow) in the control cells, but the promoter area loses the signal in the NET39 overexpression cell line.

NET39 is a muscle-specific NET that influences gene positioning and expression in myogenesis;^6^ so we sought to directly compare the gene expression and positioning changes for NET39 overexpression in HT1080 fibroblasts to changes that occur in gene expression and positioning during C2C12 *in vitro* myogenesis.^6^ In the C2C12 differentiation study, knockdown of NET39 resulted in altered expression of 20% of all genes normally changing between myoblasts and myotubes. In contrast, only 129 genes (3% of all genes normally changing during myogenesis) were affected in HT1080 cells after NET39 overexpression (Fig. 5A; Supplementary Table S3). Looked at in the opposite way, 75% of all the genes changing from their normal expression pattern upon NET39 knockdown in myogenesis were myogenic while only 20.6% of the genes (129 out of 626) altered by exogenous expression of NET39 in HT1080 fibroblasts were myogenic genes. In the C2C12 myogenesis system^6^ the function of muscle-specific NET39 slightly favored gene repression over activation (56% vs 44%). Interestingly, 75% of genes subject to repression by NET39 function in the C2C12 differentiation system were normally repressed in myogenesis and 95% of activated genes were normally activated in myogenesis. In the current study of NET39 function in the heterologous HT1080 fibroblast system a tendency toward repression by NET39 was also observed; however, the relationship with myogenic genes was, as might be expected, much weaker. In the heterologous system only 23% of the downregulated genes (100 out of 431) and 15% of the upregulated genes (29 out of 195) were myogenic (Fig. 5A). In the HT1080 cells NET39 expression was sufficient to recruit 39 of the genes that normally move toward the NE during myogenesis in the C2C12 myogenic differentiation system (Fig. 5B; Supplementary Table S3). Overall NET39 recruited more genes to the periphery (732 + 39 = 771) than away from the periphery (473 + 39 = 502), consistent with its also favoring gene repression over activation.

The transcription factor PPARγ is well known as a master regulator of adipogenesis;^36^ however, it is also an important regulator of myogenesis whose expression must be tightly modulated at the very early stages of differentiation,^37^ but is switched off afterwards.^6^ In addition to this important function, this gene was also of interest as it moved to the periphery, but rather than being repressed it was upregulated — albeit to differing degrees — in both the NET39 and NET47 expressing cells. Looking closely at the *PPARG* locus, in control HT1080 cells there is normally a small LAD covering the promoter and 50–60 Kb upstream from it, while the body of the gene is in a long stretch devoid of DamID signal (Fig. 5C). Upon overexpression of NET39, however, a LAD of about 200 Kb forms encompassing the gene body and extending around 30 Kb after the 3′ end. At the same time, a small region around the promoter seems to actually lose DamID signal, and *PPARG* is strongly upregulated (Supplementary Table S1A).

## Discussion

NET39 and other muscle-specific NETs recruit critical myogenic genes to the nuclear periphery during muscle differentiation, thus adding roughly 1/3–2/3 of their normal repression; however, ectopic expression of these NETs in myoblasts was still able to reposition the genes but without corresponding changes in gene expression.^6^ This suggested that repositioning occurs independently of the mechanism for gene regulation and then once at the nuclear periphery some as yet unidentified aspect of the periphery combined with transcriptional regulators induced during differentiation directs the gene expression changes. The nature of this functional role of the NE in myogenesis remains unclear. However, our new study clearly shows that –at least for the subset of genes identified here– tissue-specific NETs are sufficient to influence gene expression and genome organization in a completely different cell type, in a manner that is consistent with the tissue-specificity of the NET. It would seem unlikely that any transcriptional regulators involved are muscle-specific due to use of the heterologous system; however, a more widely expressed transcriptional regulator that normally functions in myogenesis could potentially act either alone or in combination with other more tissue-specific factors.

Although fewer genes were regulated by NETs in the HT1080 cells, these genes were typically those that require ‘tighter’ regulation in their natural context. When genes upregulated by NET47 in the HT1080 cells were compared with genes normally upregulated in hepatogenesis, the overlapping set included several for which the levels are altered in hepatocellular carcinoma or other liver diseases such as non-alcoholic steatohepatitis: *FBLN1*,^38^ *KLK6*,^28^ *TGFBR3*,^31^ *NDRG2*,^39^ *PCOLCE*,^32^ and *ACSL5*.^40^ Moreover, in the myogenesis study several genes induced during muscle injury were under muscle NET regulation, suggesting an additional NET function in priming genes critical for regeneration.^6^ Similarly, here the NET47-regulated genes *KLK6*,^28^ *MATN2*,^29^ and *HSPB8*^41^ play roles in responding to liver injury and regeneration. Accordingly, the liver genes negatively regulated by NET39 expression in the HT1080 fibroblasts included *TDO2* that is regulated by bone morphogenic trancsription factors in liver injury,^42^ *TBX3* that has its levels altered at multiple stages in liver differentiation,^43^ *ENDOD1* that is associated with metastasis,^44^ and *NNMT* that needs to be tightly regulated or else it can yield obesity.^45,46^

While there are many possibilities for what contributes the gene regulation in these cases, one stands out due to previous links with NE-directed gene regulation: the addition of silencing marks once at the periphery. Silencing enzymes such as HDAC3 are recruited to the periphery by the ubiquitously expressed NETs LAP2ß and emerin.^14,15^ In fact, in mouse cells Hdac3 has been shown to work cooperatively with emerin to regulate both the position and expression of 3 important myogenic genes *MyoD, Myf5* and *Pax7*.^47^ More complex interactions have been reported where multiple NE proteins work together with transcriptional regulators and silencing enzymes to promote specific peripheral tethering of the *Cyp3* and *IgH* loci in fibroblasts.^48^ Thus, the ability to combine repositioning with changes in expression likely depends on the presence of specific combinations of ubiquitous NETs plus tissue-specific ‘repositioning’ NETs.

This was likely the case for the *PPARG* upregulation with exogenous NET39 expression. PPARγ is an important transcriptional driver for adipogenesis but it also plays a role in myogenesis, where critical expression of *PPARG* is necessary for early differentiation of skeletal muscle cells: altered expression inhibits myogenesis.^37^ In our HT1080 control cell line the *PPARG* gene was mostly in an inter-LAD position, but it clearly repositioned toward the periphery in the muscle-specific NET39-expressing HT1080 line. There was a strong increase in lamin B1-DamID signal in the middle of the gene and upstream of the promoter; however this repositioning was associated with a shift that made the promoter slightly more accessible, with a window of ∼30 Kb showing no peripheral association. In C2C12 myogenesis *PPARG* was both downregulated and repositioned to the periphery.^6^ In a differentiating muscle cell there would likely be muscle-specific factors that might either modify the LAD footprint to include the promoter or else sit on the promoter to more tightly regulate the gene. However, in the NET39-expressing HT1080 line, this was the most highly upregulated gene (Supplemental Table S1A) while it was only weakly expressed in the NET29-expressing HT1080 line. Based on this separation between NE positioning and gene expression, we propose that these events involve distinct mechanisms which were uncoupled in the heterologous cell assay. In the absence of the tightly coordinated cascades that govern differentiation, the gene repositioning action of NET39 may be incomplete, perhaps due to conflicting signals. In the heterologous system NET39 expression results in activating 2 opposing processes: first, the repositioning of *PPARG* to the periphery, and, second, its upregulation. As a result, the small LAD just upstream of *PPARG* extends to include most of the gene, except for a ∼30 Kb window around the promoter. It is unclear whether this is because the cells are maintained in an ‘early differentiation’ like state, where the switch to silencing has not yet occurred and the gene is positioned in an environment conducive to quick and permanent silencing, or perhaps the switch is ineffective because the HT1080 cells do not express myoblast-specific factors.

This specific example of a gene that is strongly upregulated while actually increasing its peripheral association shows that the reported gene activation driving release from the periphery^11^ is not a universal mechanism. Further to this point the global intersects between the repositioning and expression data yielded many examples of genes becoming activated at the periphery without being released (Fig. 4A). The fact that a system as removed as a fibroblast cell line can react in such a tissue-specific manner to overexpression of these NETs highlights the importance of these NE proteins.

These data contribute to our understanding of the NE's role as an exciting additional layer of genome regulation. Multiple mechanisms can function to regulate gene expression associated with gene repositioning. Nonetheless, while the partial overlaps between the specific genes repositioned and regulated by NET39 in myogenesis and in the HT1080 fibroblasts indicate that the NETs alone contribute considerably to this mechanism, at the same time the fact that many more tissue-specific genes were affected in the tissue differentiation system argues that such questions are best addressed using actual tissues or differentiation systems. Tissue-specific NETs are clearly involved in genome organization^6,7,22^ and can be used to manipulate endogenous genes and chromosomes independently of differentiation. Future investigations of how these NETs and the genes they regulate interact in differentiation with transcriptional regulators and other partners should yield further insights into how 3D spatial genome organization contributes to gene expression regulation.

## Materials and methods

### Plasmid construction

NET-GFP fusions have been previously described^7,49^ psPAX2 and pMD2.G were a gift from Justina Cholewa-Waclaw (Adrian Bird, WTCCB, Edinburgh). pLgw Dam-V5-Lamin B1 and pLgw V5-Dam were a gift from the van Steensel laboratory.

### Cell culture and transfections

Human HT1080 fibroblasts and derivatives stably expressing NETs were maintained in high glucose DMEM supplemented with 10% fetal bovine serum (FBS), 100 µg/ml penicillin and 100 µg/ml streptomycin sulfate. HT1080 cells were stably transfected using linearized plasmids carrying NET-GFP fusions. Transfectants were initially selected for with 500 µg/ml Geneticin for 2 weeks and surviving cells were further enriched for those expressing the GFP fusions by FACS. Cells were maintained thereafter with 100 µg/ml Geneticin.

### Microarrays

Total RNA from cells stably transfected with NET-GFP fusions and from controls (transfected with NLS-GFP and untransfected) was extracted with TRIzol Reagent (Ambion, 15596026) according to the manufacturer's instructions. The RNA was converted to cRNA and labeled with biotin using the Illumina TotalPrep RNA Amplification Kit (Ambion, AMIL1791). For each analysis, at least 3 biological replicates were hybridized to Illumina whole genome gene expression arrays (HumanHT-12 BeadChip v3). These arrays have a coverage of 48,803 transcripts representing 18,187 RefSeq genes (HG19).

Hybridizations were performed by the Wellcome Trust Clinical Research Facility associated with the University of Edinburgh, using an Illumina Beadstation. Microarray data were quantile normalized and analyzed in the R environment using the Bioconductor package Limma^50^ using the NLS-GFP transfected and untransfected HT1080 samples as a reference. We selected differentially expressed transcripts using moderated F-statistics and adjusted for a false discovery rate of 5%^51^ and a log2(signal ratio) above 0.5. Data available at GEO accession GSE87228.

### Lentivirus generation and transduction

Lentiviruses encoding DamID constructs were generated as described in^52^ with several modifications. Briefly, non-replicative lentiviruses were generated by transfection of ∼6 million 293FT cells plated in a 8.5 cm diameter tissue culture plate with 2.8 μg pMD2.G, 4.6 μg psPAX2 and 7.5 μg of the construct-specific transfer vector using 36 µl lipofectamine 2000 in 3 ml Optimem as per the manufacturer's instructions. After 16 h 293FT media was replaced. 48 h later the virus containing supernatant was aspirated, cleared of cellular debris by centrifugation using Lenti-X concentrator (Clontech) according to the manufacturer's instructions and resuspended in 200 µl of Opti-MEM. If not used immediately, aliquots were frozen at −80°C. Transduction was performed in the presence of 6 μg/ml polybrene using 5–10 µl of lentiviral suspension per dish in a 6-well plate that was seeded the previous night with 1.5–2 × 10^5^ cells.

### DamID

DamID was performed as described in.^34^ Briefly for each DamID sample 3 dishes of a 6-well plate were seeded with 1.5–2×10^5^ HT1080 cells each, and 24 h later they were transduced with 5–10 µl of a Dam methylase encoding lentiviral preparation in the presence of 6 µg/ml polybrene. After 72 h cells were trypsinized, pelleted at 1,000 x *g* for 2 min and DNA extracted. DamID sample processing was then performed as described in Vogel et al. Briefly, DNA was extracted from cells using the DNeasy tissue lysis kit (Qiagen, 69504) as per manufacturer's instructions. 2.5 µg of extracted DNA was then digested by *DpnI* (NEB) and, following heat inactivation of *DpnI*, was ligated to the DamID adaptor duplex (dsAdR) generated from the oligonucleotides AdRt (5′-CTAATACGACTCACATAGGGCAGCGTGGTCGCGGCCGA-GGA-3′) and AdRb (5′-TCCTCGGCCG-3′) after which DNA was further digested by *DpnII*. To amplify DNA sequences methylated by the Dam methylase, 5 µl of *DpnII* digested material was then subjected to PCR in the supplied buffer in the presence of the 1.25 µM Adr-PCR primer (5′-GGTCGCGGCCGAGGATC-3′), 0.2 mM dNTPs and 1X of the Advantage cDNA polymerase (Clontech, cat. no. 639105). PCR was performed with 1 cycle at 68°C for 10 min followed by 1 cycle of 94°C for 3 min, 65°C for 5 min and 68°C for 15 min, then followed by 4 cycles of 94°C for 1 min, 65°C for 1 min and 68°C for 10 min, and finally followed by 17 cycles of 94°C for 1 min, 65°C for 1 min and 68°C for 2 min. Following PCR, the distribution of amplified DNA fragments was checked on agarose gels and purified on QIAquick PCR purification columns (Qiagen, 28104) and then concentrated to the required concentration by precipitation. To generate the 2 µg of material required of next generation sequencing it was found an average of 6–8 PCR reactions were required per sample.

DamID sample libraries were prepared for next generation sequencing by fragmentation followed by ligation to sequencing adaptors. DamID sequences (GEO accession GSE87228) were aligned to the human Hg19 genome using the Burrows-Wheeler Aligner software bwa-mem.^53^ Subsequent processing was performed using R (R Core Team (2015). R: A language and environment for statistical computing. R Foundation for Statistical Computing, Vienna, Austria. URL https://www.R-project.org/) and Bedtools.^54^ Data was quantified by counting the number of reads per DpnI-flanked (GATC) genomic fragment for each pair of Dam-alone and Dam-LaminB1 samples. The log2 ratios between Dam-LaminB1 and Dam-alone were calculated for each DpnI fragment, and the resulting values quantile normalized in R using the BioConductor Limma package^50^ Data shown on profiles were smoothed by substituting the value for each fragment to the average of its +/−50 nearest DpnI fragments (median ∼30Kb).

To identify LADs we used a circular binary segmentation (CBS) algorithm in the Bioconductor package DNAcopy (Seshan VE and Olshen A (2016). *DNAcopy: DNA copy number data analysis*. R package version 1.46.0) using the default parameters. The positive signal tracts were extracted and merged if they were <5 Kb apart. We removed anything below 15 Kb from our LAD list. This threshold was chosen empirically after careful examination of the LAD traces. We considered a gene being in a LAD if it at least overlapped with one.

To identify genomic regions with differential frequencies of association with the periphery between WT HT1080 cells and HT1080 cells stably expressing NETs, we used essentially the method we described in Robson et al.^6^ with small variations. Briefly, we segmented the genome into small windows and compared the signal between conditions for each window using a composite filter where a window was initially highlighted if either a) its signal was positive in one sample and negative (or absent) in the other, or b) both samples had positive signals but the one was at least twice the value of the other. Then we also calculated straight LAD differences by subtracting LAD sets and added them to the list. The resulting highlighted windows were merged if closer than 5 Kb and we discarded any smaller than 15 Kb after visual inspection to produce a set of differential regions or DRs. These regions could be then statistically tested for enrichment against a random signal distribution generated from the raw data using Fisher's tests iteratively. In the present study, we segmented the genome using the CBS algorithm mentioned earlier, instead of using fixed-width windows.

Regions that experienced an increase in signal were termed IP, representing a shift from the interior toward the periphery, while regions that experienced a decrease in signal were termed PI and represented a shift away from the periphery. Genes that overlapped IP or PI regions were referred to as IP or PI genes respectively.

### Functional analysis of gene sets

Genes differentially expressed in HT1080 and NET-expressing HT1080 cells were analyzed for Biological Process and Cellular Compartment GO-term enrichment using Gene Ontology enrichment analysis and visualization tool GOrilla.^55^ GO-terms reflecting different functional groupings that were statistically enriched were selected and manually joined (Table S1C) for the larger groupings represented in Fig. 1C. “gprofiler”^56^ was used to carry out the gene set enrichment analysis on the human genes in the ortholog groups of genes. Table S1B contains the output of results showing significant enrichment to what is expected if the genome was randomly sampled. Tables S1A, B and C are each separate worksheets in one excel file.

For Supplementary Table S3 in addition to the gene set listings there is also a transcription factor analysis performed using EnrichR.^57^

## Supplementary Material

Supplemental_Materials.zip

## Abbreviations

NE: nuclear envelope
NET: nuclear envelope transmembrane protein.
