# Supplementary material for: Tissue-specific NETs alter genome organization and regulation even in a heterologous system
Source: Nucleus. 2017 Jan 3;8(1):81–97. doi: 10.1080/19491034.2016.1261230 (PMC5287206; doi:10.1080/19491034.2016.1261230)
Supplement: Supplemental_Materials.zip [file 2016NUCLEUS0016R1-s01.pdf › 2016NUCLEUS0016R1-s01.pdf]

November 2, 2016

Eric C. Schirmer, Group Leader
WELLCOME TRUST CENTRE *for* CELL BIOLOGY
Institute of Cell Biology, The University of Edinburgh
5.22 Michael Swann Building, Max Born Crescent
Edinburgh EH9 3BF, Scotland, UK

Prof. Iris Meier
Associate Editor
Nucleus

Re: Special Issue Submission 2016NUCLEUS0016

Dear Professor Meier,

Please find attached our second revision of 2016NUCLEUS0016 for the special issue of *Nucleus* devoted to the *2016 Society for Experimental Biology* meeting. We have addressed all of the reviewer comments. We hope it will now be deemed acceptable for publication in *Nucleus*.

Sincerely,

Eric C. Schirmer, Ph.D.
Wellcome Trust Senior Research Fellow
The Wellcome Trust Centre for Cell Biology,
and Reader, Institute of Cell Biology, and
Centre for Translational and Chemical Biology
University of Edinburgh, Kings Buildings
Michael Swann Building, room 5.22, Max Born Crescent
Edinburgh, EH9 3BF, UK
+44 131 650-7075 (office), -7095 (lab), -7360 (fax)
e-mail: e.schirmer@ed.ac.uk
http://www.wcb.ed.ac.uk/research/schirmer
